# Sex steroids differentially modulate SARS-CoV-2-induced inflammatory responses in endothelial and immune cells

**DOI:** 10.3389/fcimb.2026.1976945

**Published:** 2026-10-14

**Authors:** Sandra Bižić-Radulović, Tijana Subotički, Ana Kovačević, Emilija Živković, Olga Popović, Olivera Mitrović-Ajtić, Miloš Diklić, Snežana Jovanović, Vladan P. Čokić, Bojana Beleslin-Čokić

**Affiliations:** 1 Department of Molecular Oncology, Clinic of Hematology, University Clinical Center of Serbia, Belgrade, Serbia; 2 Department of Molecular Oncology, Institute for Medical Research, National Institute of Republic of Serbia, University of Belgrade, Belgrade, Serbia; 3 Department of Molecular Oncology, Institute of Virology, Vaccines and Sera “Torlak”, Belgrade, Serbia; 4 Department of Microbiology, University Clinical Centre of Serbia, Belgrade, Serbia

**Keywords:** dihydrotestosterone, estradiol, immune cells, inflammatory signaling pathways, SARS-CoV-2

## Abstract

**Introduction:**

Sex-related differences in coronavirus disease 2019 (COVID-19) severity suggest an important role for sex steroids in regulating immune and endothelial responses to severe acute respiratory syndrome coronavirus 2 (SARS-CoV-2).

**Methods:**

Receptor expression, inflammatory signaling, and monocyte transendothelial migration were assessed by RT-PCR, immunoblotting, and Boyden chamber assay, respectively. HULEC-5a and primary human immune cells were exposed to *in vitro*-amplified SARS-CoV-2. Samples were collected between June 2020 and April 2022, with sample sizes based on primary sample availability and independent experiments.

**Results:**

SARS-CoV-2 differentially regulates estrogen receptor gene expression, decreasing it in endothelial cells (p<0.05) while increasing it in immune cells (p<0.01). SARS-CoV-2 generally activated inflammatory signaling pathways, including NFκB, STAT3, and p38 MAPK, in THP-1 monocytic cells (up to 1.9-fold). The NFκB response was augmented by the androgen receptor agonist dihydrotestosterone (DHT, 44.6%) and attenuated by the androgen receptor antagonist flutamide (44.1%). In endothelial cells, DHT twice reduced SARS-CoV-2 activation of the p38 MAPK signaling pathway. β-estradiol inhibited (2.2-fold), whereas DHT stimulated (4.9-fold) NFκB signaling pathway in neutrophils isolated from patients with COVID-19. SARS-CoV-2 activation of inflammatory signaling pathways deteriorates over time in monocytes from patients with COVID-19 (p<0.05). Furthermore, DHT significantly increased trans-endothelial migration of monocytes (p<0.001) and detachment of SARS-CoV-2 exposed endothelial cells (p<0.01), suggesting a potential role for androgen-responsive pathways in endothelial dysfunction and immune cell trafficking.

**Discussion:**

These findings elucidate the molecular mechanisms driving male-biased COVID-19 severity and underscore the potential of targeting androgen-responsive pathways as a therapeutic strategy to mitigate severe viral inflammation and endothelial dysfunction.

## 1 Introduction

Biological sex significantly influences the clinical progression of COVID-19. Reduced testosterone levels in male patients are consistently associated with severe disease manifestations, such as hypoxemic respiratory failure, hyperinflammation, and increased mortality (Vena et al., 2022; Infante et al., 2021; Lanser et al., 2021; Dhindsa et al., 2021; Camici et al., 2021). Individuals with severe COVID-19 present with markedly lower testosterone concentrations compared to those with milder forms, and testosterone levels inversely correlate with pro-inflammatory cytokines, particularly IL-6 (Infante et al., 2021; Dhindsa et al., 2021; Camici et al., 2021). A progressive decline in testosterone, accompanied by elevated IL-10 levels, is further linked to increased disease severity and mortality risk (Amodio et al., 2023). Conversely, elevated estradiol-to-testosterone ratios are associated with hyperinflammatory states and higher hospital mortality (Infante et al., 2021). SARS-CoV-2 infection also disrupts reproductive hormone profiles, resulting in increased luteinizing hormone (LH) and follicle-stimulating hormone (FSH) levels (Enikeev et al., 2022). Moreover, estradiol levels are inversely related to the duration of viral shedding during recovery (Xu et al., 2021). Beyond COVID-19, sex hormones play a central role in the pathogenesis of numerous immune-mediated diseases. Androgen and estrogen signaling contribute to the sex bias observed in autoimmune disorders such as systemic lupus erythematosus, rheumatoid arthritis, and multiple sclerosis, as well as in cardiovascular disease, metabolic syndrome, and several viral infections (Mauvais-Jarvis et al., 2020).

Building on these clinical observations, experimental studies have demonstrated that testosterone and estradiol exert distinct immunomodulatory effects during SARS-CoV-2 infection. Estradiol treatment reduces inflammatory biomarkers, including IL-6, in hospitalized COVID-19 patients (Lovre et al., 2024), and estrogen signaling suppresses virus-induced inflammatory and immune responses (Li et al., 2022). In cellular models, both testosterone and estradiol modulate macrophage inflammatory responses to COVID-19-associated stimuli *in vitro* (Allahverdiyeva et al., 2024), with effects dependent on cell type and experimental context. Estradiol reduces inflammation in lung epithelial models of COVID-19-associated hyperinflammation (Zhu et al., 2022), whereas testosterone enhances monocyte pro-inflammatory responses by increasing TNF, IL-6, and NFκB-regulated signaling (Lakshmikanth et al., 2024). Furthermore, dihydrotestosterone exacerbates endothelial injury and monocyte adhesion induced by the SARS-CoV-2 spike protein (Kumar et al., 2021), indicating a potential role for androgens in thromboinflammatory processes.

Endothelial dysfunction, immune hyperactivation, and thrombus formation are major contributors to severe COVID-19 morbidity and mortality. However, how sex steroids modulate inflammatory responses in endothelial and immune cells during SARS-CoV-2 infection remains insufficiently characterized. We hypothesized that β-estradiol and dihydrotestosterone (DHT) exert opposing effects on inflammatory signaling and monocyte–endothelial interactions during SARS-CoV-2 exposure. We therefore analyzed the expression of selected estrogen and androgen receptors in endothelial and immune cells and investigated the effects of β-estradiol and DHT, with or without SARS-CoV-2 exposure, on NF-κB, STAT3, and p38 MAPK signaling in endothelial cells, monocytes, and neutrophils. Transendothelial monocyte migration was additionally assessed as a functional model linking sex hormone-dependent vascular inflammation with leukocyte recruitment and processes relevant to thromboinflammation.

## 2 Materials and methods

### 2.1 Isolation and amplification of SARS-CoV-2

Peripheral blood samples and nasopharyngeal swabs from patients with COVID-19, confirmed by positive RT-PCR testing in the Department of Microbiology, University Clinical Center of Serbia (UCCS), were collected at the Clinic for Infectious and Tropical Diseases, UCCS, Belgrade, Serbia, between June 2020 and April 2022. The study was conducted in accordance with the Declaration of Helsinki and approved by the Ethics Committee of the University Clinical Center of Serbia (protocol code 570/12) and the Institute for Medical Research (131/2020). Written informed consent was obtained from all patients. VERO C1008 cells (ECACC, Cat. No. 85020206, Culture Collections UK Health Security Agency, Salisbury, UK) were inoculated with the Wuhan-1 SARS-CoV-2 variant obtained from a nasopharyngeal swab of a patient with confirmed COVID-19. Viral identity was confirmed by sequencing analysis as previously described (Bižić-Radulović et al., 2025). After propagation, the same viral stock was used for all subsequent experiments. First, the DMEM medium (Sigma-Aldrich, Inc., St. Louis, MO, USA) was decanted, and then the viral inoculum was added to the VERO C1008 cells. The cells with SARS-CoV-2 were incubated for 30 min at 37 °C, after which we added growth medium (DMEM with 2% FBS; PAN-Biotech GmbH, P40-37500, Aidenbach, Germany) to the desired volume without prior removal of the viral suspension to propagate the virus. During virus passage, we performed Applied Biosystems QuantStudio 5 Real-Time qPCR (Thermo Fisher Scientific, Waltham, MA, USA) at each step to confirm SARS-CoV-2 presence, and at the end, a 50% Tissue Culture Infectious Dose (TCID50) titration assay was performed. Cell infection was confirmed by the presence of cytopathic effects (CPE; Primovert, Carl Zeiss Microscopy, Oberkochen, Germany). SARS-CoV-2 samples were sterile/filtered and aliquoted in DMEM with 2% FBS (PAN-Biotech GmbH), and 1% Penicillin—Amphotericin B (Biowest, Lakewood Ranch, FL, USA). To determine viral titer and infectious viruses, we measured plaque-forming units (PFU)/mL via plaque assay, which was used to determine the multiplicity of Infection (MOI). Inoculations with SARS-CoV-2 into human blood cells (neutrophils and monocytes) and male cell line cultures (lung microvascular endothelium HULEC-5a and THP-1 NF-ƙB-Luc2 monocytic leukemia cell lines, ATCC, Manassas, VA, USA) were done at MOI = 0.001, for 24 hours (h) and 48 h of incubation. All procedures involving the isolation and propagation of live SARS-CoV-2 were conducted in a certified Biosafety Level 3 (BSL-3) laboratory in accordance with institutional biosafety regulations and World Health Organization (WHO) recommendations for laboratory biosafety related to SARS-CoV-2 (World Health Organization, 2021).

### 2.2 Cell cultures

This was an *in vitro* experimental study using two human male cell lines: HULEC-5a and THP-1 NF-ƙB-Luc2 monocytic leukemia cells (THP-1), as well as primary monocytes and neutrophils isolated from the peripheral blood of COVID-19 patients. Peripheral blood was processed by density gradient centrifugation (LSM, Capricorn Scientific GmbH, Ebsdorfergrund, Germany). Further, a Percoll gradient (1130 g/mL, Sigma-Aldrich) was used to isolate monocytes from the mononuclear layer, followed by hypotonic lysis of the precipitated erythrocytes to isolate the pelleted neutrophils. All cell types were cultured in 6-well culture plates (NUNC, Thermo Fisher Scientific, Waltham, MA, USA) in RPMI-1640 medium (Biowest, Nuaille, France) containing 10% FBS (Biowest) under standard conditions: 37 °C, 5% CO_2_, and 95% humidity. HULEC5a (5 × 10^5^) cells were treated with androgen receptor (AR) agonist DHT (100 nM, 5α-Androstan-17β-ol-3-one, Sigma-Aldrich) or antagonist flutamide (1 μM, Sigma-Aldrich). After 30 min of hormone or antagonist preincubation, HULEC-5a were exposed to the propagated SARS-CoV-2 Wuhan-1 viral stock, described above, at MOI = 0.001, with viral input calculated from TCID_50_ values using a correction factor of 0.7. THP-1 cells (3 × 10^6^) were exposed to DHT (10 and 100 nM) and flutamide (100 nM and 1 μM) and than exposed to SARS-CoV-2 at MOI = 0.001. The cultures of primary monocytes and neutrophils, of independent biological replicates, were also treated with 1 nM, 10 nM, and 100 nM of DHT and β-estradiol (Tocris Bioscience, Bristol, UK), with or without virus treatment. The sex steroids and antiandrogen were dissolved in dimethyl sulfoxide (DMSO), and the corresponding DMSO vehicle control was used for each treatment condition. Following 24 h and 48 h of incubation, HULEC-5a and THP-1, as well as primary monocytes and neutrophils (0.5–6 × 10^6^ cells), were harvested for gene expression analysis by RT-qPCR and signaling pathways analysis by Western blot.

### 2.3 Real time quantitative PCR

Total RNA was isolated by using the TRIzol™ reagent (Bioline Ltd., London, UK) from monocytes/neutrophiles and HULEC-5a/THP-1 cells, according to manufacturer’s protocol. Concentration and integrity of total RNA were assessed by Nanophotometer P330 (Implen GmbH, München, Germany). Equal amounts of RNA from different samples were transcribed into cDNA using the High-Capacity cDNA Reverse Transcription Kit with RNase Inhibitor (Thermo Fisher Scientific) according to the manufacturer’s instructions. Quantitative PCR was performed using Amplifyme SYBR Universal Mix, Blirt (Gdańsk, Poland) on a Magnetic Induction Cycler (Bio Molecular Systems, Upper Coomera, QLD, Australia). Sequences for primers used for analysis were obtained from Origene (Rockville, MD, USA). All primers were synthesized by Integrated DNA Technologies (Coralville, IA, USA), the sequences are listed in **Table 1**. PCR reaction conditions consisted of 50 °C for 2 min and 95 °C for 2 min, followed by cycling between a melting temperature of 95 °C for 3 s, an annealing temperature of 60 °C for 30 s, and extension temperature of 72 °C for 30 s repeated for 40 cycles, followed by melting curve analyses. For statistical significance, two technical repeats were used for analysis of target genes and human *β actin* was used as a reference gene. Relative expression of the targeted gene was calculated by the 2^−ΔΔCt^ method.

**Table 1:** Primer sequences.

Primer	Sequence (5′–3′)
*ESR1* forward	GCTTACTGACCAACCTGGCAGA
*ESR1* reverse	GGATCTCTAGCCAGGCACATTC
*AR* forward	ATGGTGAGCAGAGTGCCCTATC
*AR* reverse	ATGGTCCCTGGCAGTCTCCAAA
*β-Actin* forward	AGC CTC GCC TTT GCC GA
*β-Actin* reverse	CTG GTG CCT GGG GCG

Estrogen receptor 1 (ESR1) and androgen receptor (AR).

### 2.4 Western blot

Whole cell extracts were prepared from cell pellets of HULEC5a, THP-1, monocytes, and neutrophils using RIPA buffer (1 mM EDTA, 50 mM Tris-HCl, pH 7.5, 0.1% SDS, 150 mM NaCl, 1% NP40, 1% sodium deoxycholate, protease inhibitor cocktail). Homogenates were separated into polyacrylamide gels and blotted to polyvinylidene difluoride membranes (GE Healthcare, Chicago, IL, USA). Signal has been detected using antibodies directed against phospho-NFκB (Ser536) (E-AB-70335, Elabscience, Houston, TX, USA), total NFκB (E-AB-22066, Elabscience), phospho-p38 (Tyr180/Tyr182) (E-AB-21027, Elabscience), total p38 (E-AB-66279, Elabscience), STAT3 (E-AB-22166, Elabscience), and pSTAT3 Tyr705 (E-AB-51040, Elabscience) and incubated overnight at 4 °C (dilution 1:1000 for all antibodies). Secondary antibodies conjugated to horseradish peroxidase (Elabscience) were detected using an enhanced chemiluminescence detection system (BioRad Laboratories, Hercules, CA, USA). Protein bands were visualized using a ChemiDoc Imager (BioRad Laboratories) and densitometric analysis was performed using Image Lab software (Bio-Rad Laboratories, Version 6.0.0.25). Band intensities were quantified from non-saturated exposures and normalized by calculating the ratio of phosphorylated protein signal intensity to the corresponding total protein signal. Quantification values were obtained from independent biological replicates and expressed relative to the untreated control group.

### 2.5 Trans-endothelial migration

A 24-well cell migration assay (CBA-102, Cell Biolabs, Inc., San Diego, CA, USA) was used to assess the migration of monocytes from SARS-CoV-2 patients across an endothelial barrier into the lower chamber. HULEC-5a cells were seeded as a confluent monolayer on 4 μm pore membranes in the upper compartment of a Boyden chamber. The cells were treated with 100 nM DHT, 100 nM β-estradiol, 1 μM flutamide, and exposed to SARS-CoV-2 at MOI = 0.001. After 24 h, monocytes (5 × 10^5^ cells/well) were added and co-cultured with the HULEC-5a monolayer for an additional 24 h. Migrated monocytes, and detached HULEC-5a, in the lower chamber were labelled with CyQuant GR dye, and fluorescence was measured using a Perkin Elmer Wallac 1420 Victor2 microplate reader (Lianstriant, UK) at excitation/emission wavelengths of 480/520 nm.

### 2.6 Statistical analysis

Sample size was determined by the availability of patient-derived cells. Normality was assessed using the Shapiro–Wilk and Kolmogorov–Smirnov tests. Differences between groups were analysed using the Student’s t-test and one-way ANOVA with Dunnett’s multiple comparisons test. When the distribution was not normal, the Mann–Whitney test was used for intergroup comparisons. All statistical analyses were performed using Prism 9 (GraphPad Software, Inc., San Diego, CA, USA). The results are expressed as mean ± SEM. The level of significance was 5%.

## 3 Results

### 3.1 Regulation of sex steroid receptor gene expression by SARS-CoV-2 in endothelial and immune cells

To investigate the role of sex steroids during SARS-CoV-2 infection, we first examined estrogen receptor 1 (*ESR1*) and *AR* gene expression in endothelial cells and immune cells. SARS-CoV-2 reduced *ESR1* expression in HULEC-5a endothelial cells after 24 h (*p* < 0.05, **Figure 1A**). In neutrophils, SARS-CoV-2 reduced *AR* expression after 24 h (*p* < 0.001), while increasing *ESR1* expression after both 24 h and 48 h (*p* < 0.05, **Figure 1B**). In monocytes, SARS-CoV-2 increased expression of both *AR* (*p* < 0.05) and *ESR1* (*p* < 0.01) after 24 h (**Figure 1C**). These findings indicate differential regulation of sex steroid receptor expression by SARS-CoV-2, characterized by reduced *ESR1* expression in endothelial cells and increased *ESR1* expression in immune cells, whereas *AR* expression was inversely regulated in neutrophils and monocytes.

**Figure 1:**
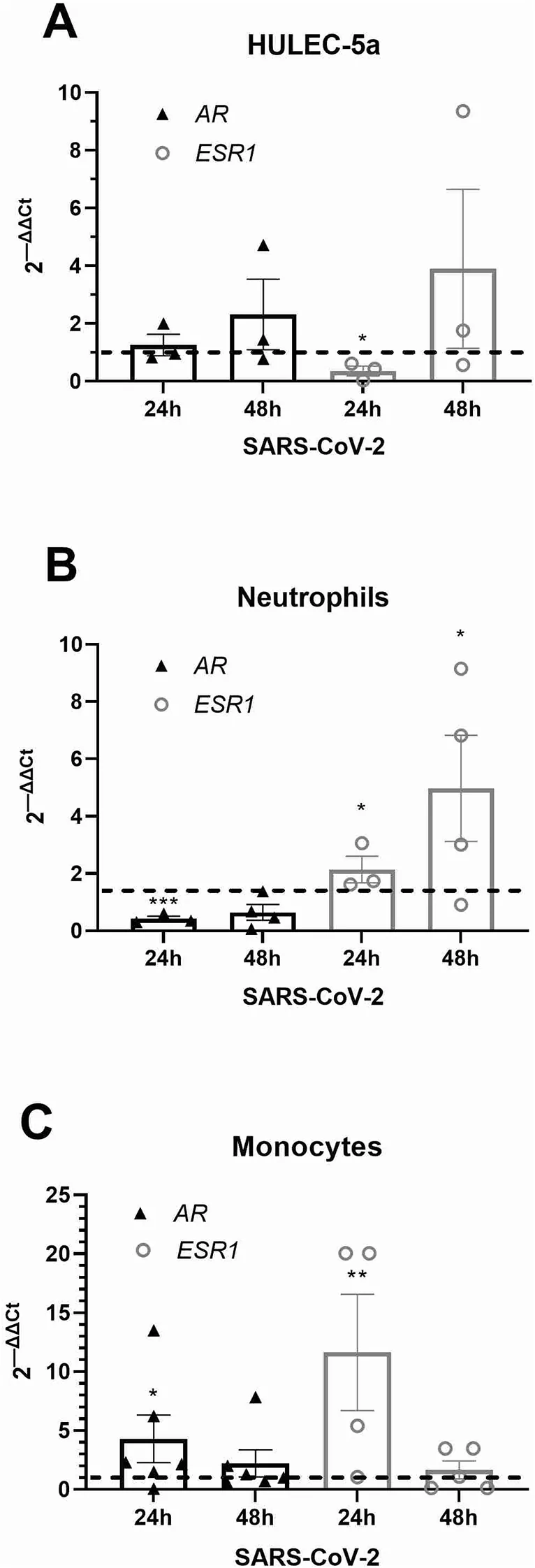
Gene expression of estrogen and androgen receptors in endothelial and immune cells exposed to SARS-CoV-2. HULEC-5a as well as primary monocytes and neutrophils, isolated from patients with COVID-19, were treated with SARS-CoV-2 (MOI = 0.001) and gene expression of estrogen (*ESR1*) and androgen (*AR*) receptors was examined by RT-qPCR after 24 h and 48 h of incubation in **(A)** HULEC-5a; **(B)** neutrophils and **(C)** monocytes. Dashed line indicates the untreated control condition. Human *β actin* was used as a reference gene and non-treated cells as the Control. Values are mean ± SEM (*n* = 3–5). *p < 0.05, **p < 0.01, ***p < 0.001 vs. Control.

### 3.2 Modulation of inflammatory signaling pathways by sex steroids and SARS-CoV-2 in monocytic cells

Because hyperinflammation is a hallmark of SARS-CoV-2 infection, we investigated whether inflammatory signaling is influenced by sex steroids using THP-1 monocytic cells. SARS-CoV-2 induced NFκB activation after 48 h treatment of THP-1 cells (*p* < 0.001), which was further increased by DHT (*p* < 0.001), whereas the AR antagonist flutamide had no significant effect (**Figure 2A**). STAT3 signaling was also activated by SARS-CoV-2 (*p* < 0.01, **Figure 2B**). This activation was not additionally enhanced by DHT but was attenuated by flutamide after 24 h (*p* < 0.001, **Figure 2B**). After 48 h, the pSTAT3/STAT3 ratio was reduced in SARS-CoV-2-exposed cells, while DHT and flutamide treatments maintained a higher ratio compared with SARS-CoV-2 alone (*p* < 0.001, **Figure 2B**). Notably, the DHT-associated increase at 48 h was 3.4-fold lower than at 24 h, whereas the flutamide-associated level was unchanged between 24 h and 48 h (**Figure 2B**). Thus, both AR activation and AR blockade were associated with sustained STAT3 phosphorylation at 48 h, but with different temporal dynamics. SARS-CoV-2 also activated p38 MAPK after 24 h (*p* < 0.05), with no additional effects observed for DHT or flutamide (**Figure 2C**). Prolonged p38 MAPK activation was maintained by a low dose of DHT (10 nM) and reduced by low dose flutamide (100 nM), but not by higher flutamide concentrations (**Figure 2D**). Overall, SARS-CoV-2 induced NFκB and STAT3 inflammatory signaling was enhanced by AR activation and reduced by AR inhibition.

**Figure 2:**
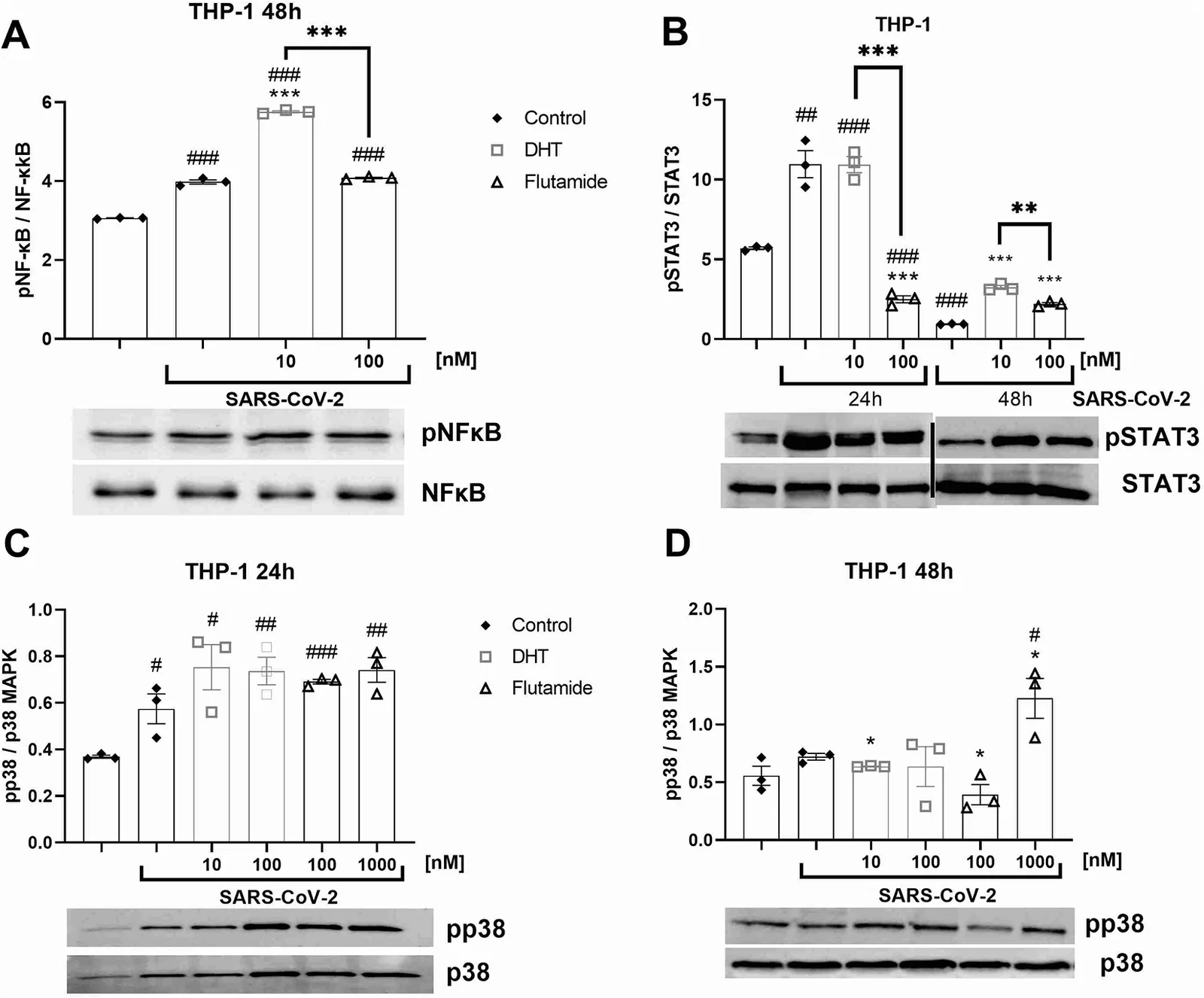
Activation of inflammatory signalling pathways in male THP-1 monocytic leukemia cell line. THP-1 cells were treated *in vitro* with dihydrotestosterone (DHT, 10 and 100 nM) and flutamide (100 and 1000 nM), in combination with SARS-CoV-2 (MOI = 0.001), and activation of **(A)** NFκB; **(B)** STAT3; and **(C, D)** p38 MAPK signalling pathways after 24 h and 48 h were examined in comparison to DHT and flutamide non-treated THP-1 cells (Control). Representative immunoblots from one independent experiment are shown. Quantitative bar graphs represent the mean ± SEM of densitometric analyses from 3 independent biological experiments after normalization of phosphorylated proteins to the corresponding total protein bands. ^#^*p* < 0.05, ^##^*p* < 0.01, ^###^*p* < 0.001 vs. SARS-CoV-2 untreated cells (Control); *p < 0.05, **p < 0.01, ***p < 0.001 vs. SARS-CoV-2 only treated cells (Control) or as indicated.

### 3.3 Modulation of inflammatory signaling pathways by sex steroids and SARS-CoV-2 in endothelial cells

Endothelial cells play a central role in inflammatory responses and coagulation processes (Mussbacher et al., 2019). Therefore, we investigated inflammatory signaling in HULEC-5a lung endothelial cells of human male origin. SARS-CoV-2 reduced STAT3 activation in HULEC-5a cells at 24 h, and this effect was not further modified by DHT or flutamide (p < 0.05, **Figure 3A**). After 48 h, DHT alone reduced STAT3 activation in the absence of SARS-CoV-2 (p < 0.01, **Figure 3B**), indicating a direct effect of androgen signaling on baseline STAT3 activity in endothelial cells. In SARS-CoV-2-exposed cells, DHT reduced virus-induced activation of p38 MAPK after 24 h (p < 0.05, **Figure 3C**). These results suggest that androgen signaling can partially suppress inflammatory signaling in endothelial cells, both at baseline (STAT3) and during SARS-CoV-2 exposure (p38).

**Figure 3:**
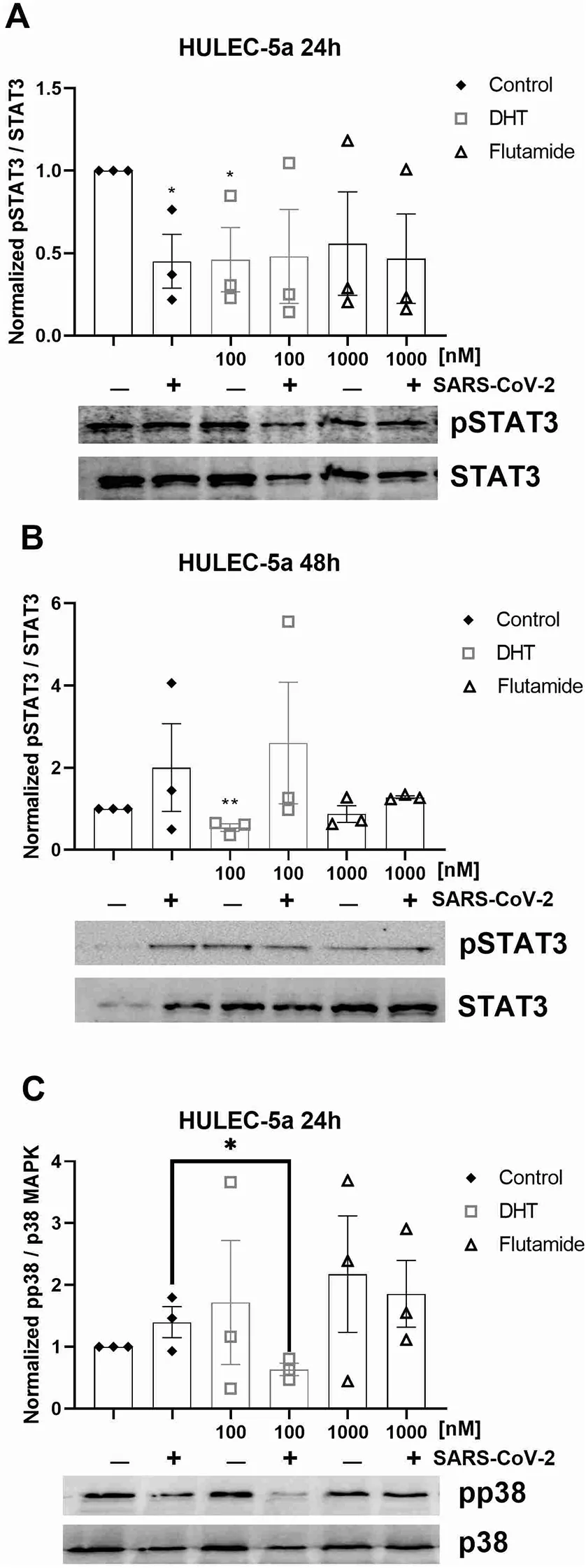
Activation of inflammatory signalling pathways in male lung microvascular endothelium HULEC-5a cell line. Lung microvascular endothelial cells (HULEC-5a) were treated *in vitro* with dihydrotestosterone (DHT, 100 nM) and flutamide (1000 nM), in combination with SARS-CoV-2 (MOI = 0.001), and activation of STAT3 signalling pathway after **(A)** 24 h and **(B)** 48 h; and **(C)** p38 MAPK signalling pathway after 24 h were examined in comparison to non-treated HULEC-5a cells (Control). Representative immunoblots from one independent experiment are shown. Quantitative bar graphs represent the mean ± SEM of densitometric analyses from 3 independent biological experiments after normalization of phosphorylated proteins to the corresponding total protein bands (expressed relative to the untreated control group). *p < 0.05, **p < 0.01 vs. untreated cells (Control) or as indicated.

### 3.4 Modulation of inflammatory signaling pathways by sex steroids and SARS-CoV-2 in neutrophils from patients with COVID-19

Neutrophils are major contributors to severe COVID-19 pathogenesis, and neutrophilia and elevated neutrophil-to-lymphocyte ratios (NLR) associated with adverse outcomes (Cesta et al., 2023). To investigate sex steroid-dependent inflammatory regulation, neutrophils isolated from patients with COVID-19 were treated with DHT or β-estradiol, with or without SARS-CoV-2, for 24 h and 48 h. Only conditions and time points with statistically significant effects are described below and shown in **Figure 4**; all other conditions were not significantly different. SARS-CoV-2 reduced NFκB activation in neutrophils from female patients after 24 h (p < 0.001, **Figure 4A**). At this time point, β-estradiol further reduced NFκB activation in a dose-dependent manner, irrespective of viral exposure (p < 0.01, **Figure 4A**), whereas no significant effect was observed at 48 h (not shown). In contrast, DHT increased NFκB activation after 48 h, independently of SARS-CoV-2 (**Figure 4B**), with no significant effect in patients with COVID-19 at 24 h (not shown). SARS-CoV-2 strongly induced p38 MAPK activation (>10-fold) after 24 h (p < 0.001, **Figure 4C**). These findings suggest that β-estradiol suppresses, whereas DHT enhances, NFκB signaling in neutrophils from patients with COVID-19.

**Figure 4:**
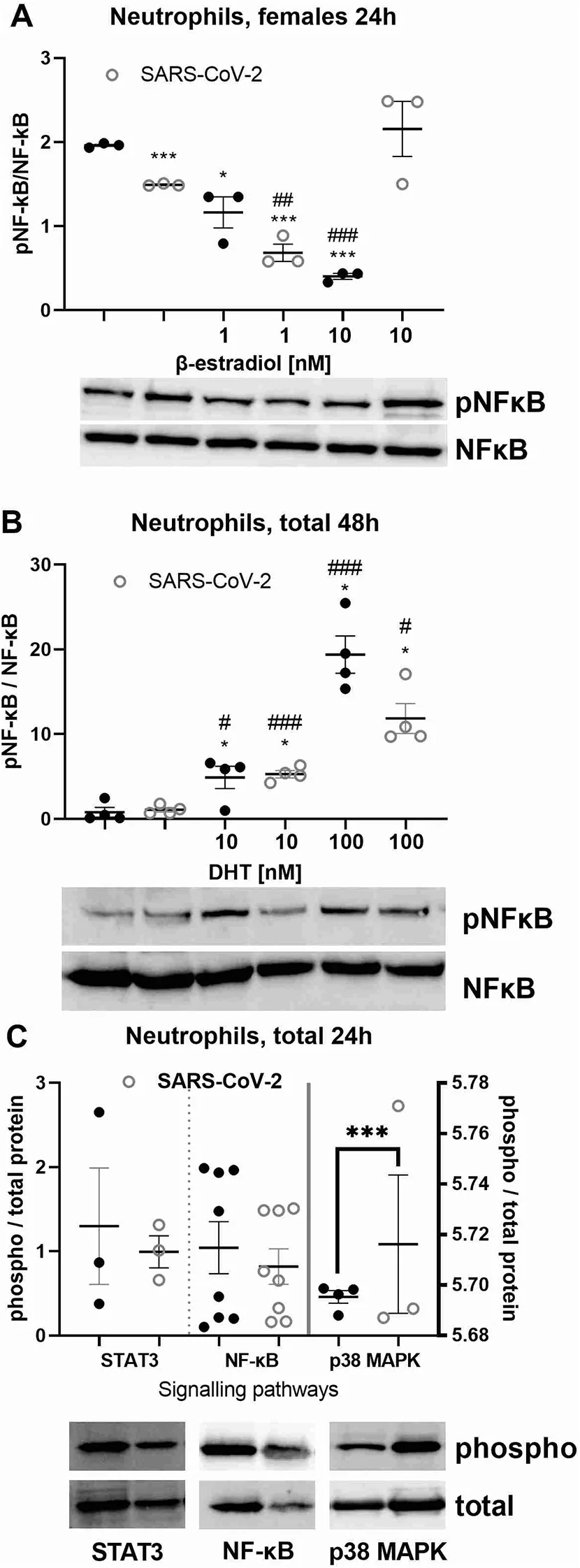
Activation of inflammatory signalling pathways in neutrophils of patients with COVID-19. Neutrophils were isolated from patients with COVID-19 and activation of NFκB signalling pathway was examined in **(A)** female patients after 24 h of incubation with β-estradiol (1 and 10 nM) and SARS-CoV-2 (MOI = 0.001, *n* = 3); **(B)** total patients after 48 h of incubation with dihidrotestosteron (DHT, 10 and 100 nM) and SARS-CoV-2 (MOI = 0.001, *n* = 4); and **(C)** activation of STAT3 (*n* = 3) and NFκB (*n* = 8) linked to left *Y*-axis as well as p38 MAPK (*n* = 4, linked to right *Y*-axis) signalling in total patients with or without SARS-CoV-2 (MOI = 0.001) after 24 h. White bars in **(A, B)** represent non-treated cells. The signaling pathways are separated by dashed and black lines as independent observations. Representative immunoblots are shown for each signaling pathway and correspond to one independent experiment. Quantitative bar graphs represent the mean ± SEM of densitometric analyses from independent biological experiments after normalization of phosphorylated proteins to the corresponding total protein bands. Comparisons were made only within the corresponding experimental dataset. *p < 0.05, ***p < 0.001 vs. untreated neutrophils or as indicated. ^#^*p* < 0.05, ^##^*p* < 0.01, ^###^*p* < 0.001 vs. SARS-CoV-2 only treated cells.

### 3.5 Modulation of inflammatory signaling pathways by sex steroids and SARS-CoV-2 in monocytes of patients with COVID-19

Monocytes are critical mediators of innate immune responses and rapidly infiltrate tissues to drive inflammation during viral infection (Mussbacher et al., 2019; Shi and Pamer, 2011). As for neutrophils, monocytes isolated from patients with COVID-19 were treated with DHT or β-estradiol (1, 10, and 100 nM), with or without SARS-CoV-2, for 24 h and 48 h. Only conditions and time points with statistically significant effects are described below and shown in **Figure 5**; all other conditions were not significantly different. In the presence of SARS-CoV-2, DHT at 1 nM enhanced NFκB activation after 24 h (p < 0.05, **Figure 5A**), whereas β-estradiol at 100 nM increased p38 MAPK activation (p < 0.01, **Figure 5B**). Moreover, β-estradiol at the lowest concentration tested (1 nM) increased p38 MAPK activation after 24 h, irrespective of viral exposure (p < 0.05, **Figure 5B**). Comparison of exposure durations showed stronger SARS-CoV-2-induced activation of NFκB and p38 MAPK after 24 h than after 48 h (p < 0.05, **Figure 5C**). These data indicate a transient but pronounced activation of NFκB and p38 MAPK signaling in monocytes during early SARS-CoV-2 exposure.

**Figure 5:**
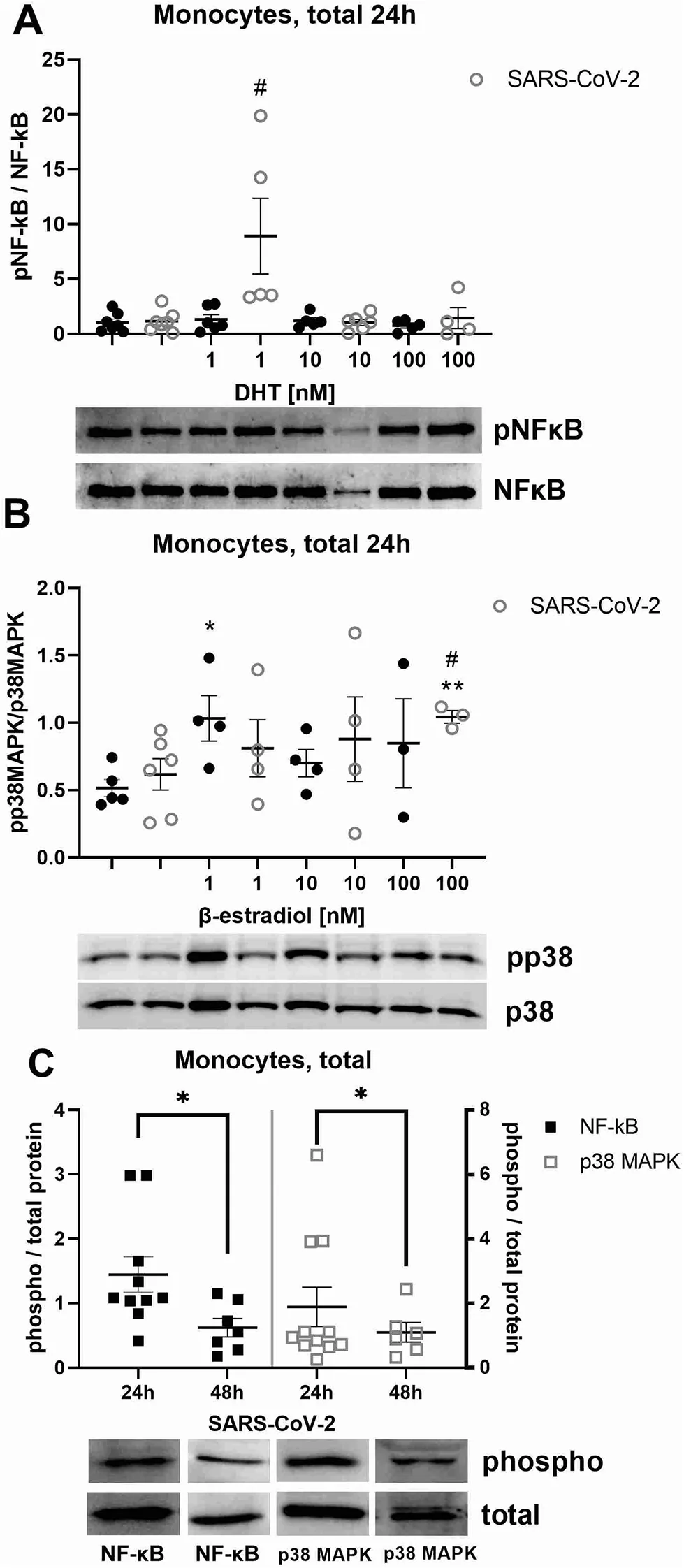
Activation of inflammatory signalling pathways in monocytes of patients with COVID-19. Monocytes were isolated from patients with COVID-19 and activation of NFκB signalling pathway was examined in total patients **(A)** after 24 h of incubation with dihidrotestosteron (DHT, 1, 10 and 100 nM) and SARS-CoV-2 (MOI = 0.001, *n* = 5–7); **(B)** after 24 h of incubation with β-estradiol (1, 10 and 100 nM) and SARS-CoV-2 (MOI = 0.001, *n* = 4–6); and **(C)** activation of NFκB and p38 MAPK signalling (linked to left and right *Y*-axis, respectively) with SARS-CoV-2 (MOI = 0.001) after 24 h (*n* = 10–11) and 48 h (*n* = 6–7). White bars in **(A, B)** represent non-treated cells. The signaling pathways are separated by a black line as independent observations. Representative immunoblots are shown for each signaling pathway and correspond to one independent experiment at the presented time points. Quantitative bar graphs represent the mean ± SEM of densitometric analyses from *n* independent biological experiments after normalization of phosphorylated proteins to the corresponding total protein bands, and were not used for direct comparison across different assembled blot panels. *p < 0.05, **p < 0.01 vs. untreated monocytes or as indicated. ^#^*p* < 0.05 vs. SARS-CoV-2 only treated cells.

### 3.6 Trans-endothelial migration of monocytes in patients with COVID-19

Trans-endothelial migration of monocytes contributes to hyperinflammation and tissue infiltration in COVID-19 (Scott et al., 2023). Using a Boyden chamber assay, we assessed monocyte migration across SARS-CoV-2 exposed endothelial monolayers. Initially, DHT increased detachment of SARS-CoV-2 exposed HULEC-5a cells after 24 h compared with SARS-CoV-2 treatment alone (*p* < 0.01, **Figure 6A**). Monocyte migration through SARS-CoV-2 exposed endothelial layers was predominantly enhanced by DHT and, to a lesser extent, by β-estradiol and flutamide (*p* < 0.001, **Figure 6B**). After correction for endothelial background fluorescence and monocyte-only controls, increased migration remained evident, particularly in DHT-treated conditions (**Figures 6C, D**). These results suggest that androgen signaling promotes endothelial dysfunction and facilitates monocyte transmigration during SARS-CoV-2 infection.

**Figure 6:**
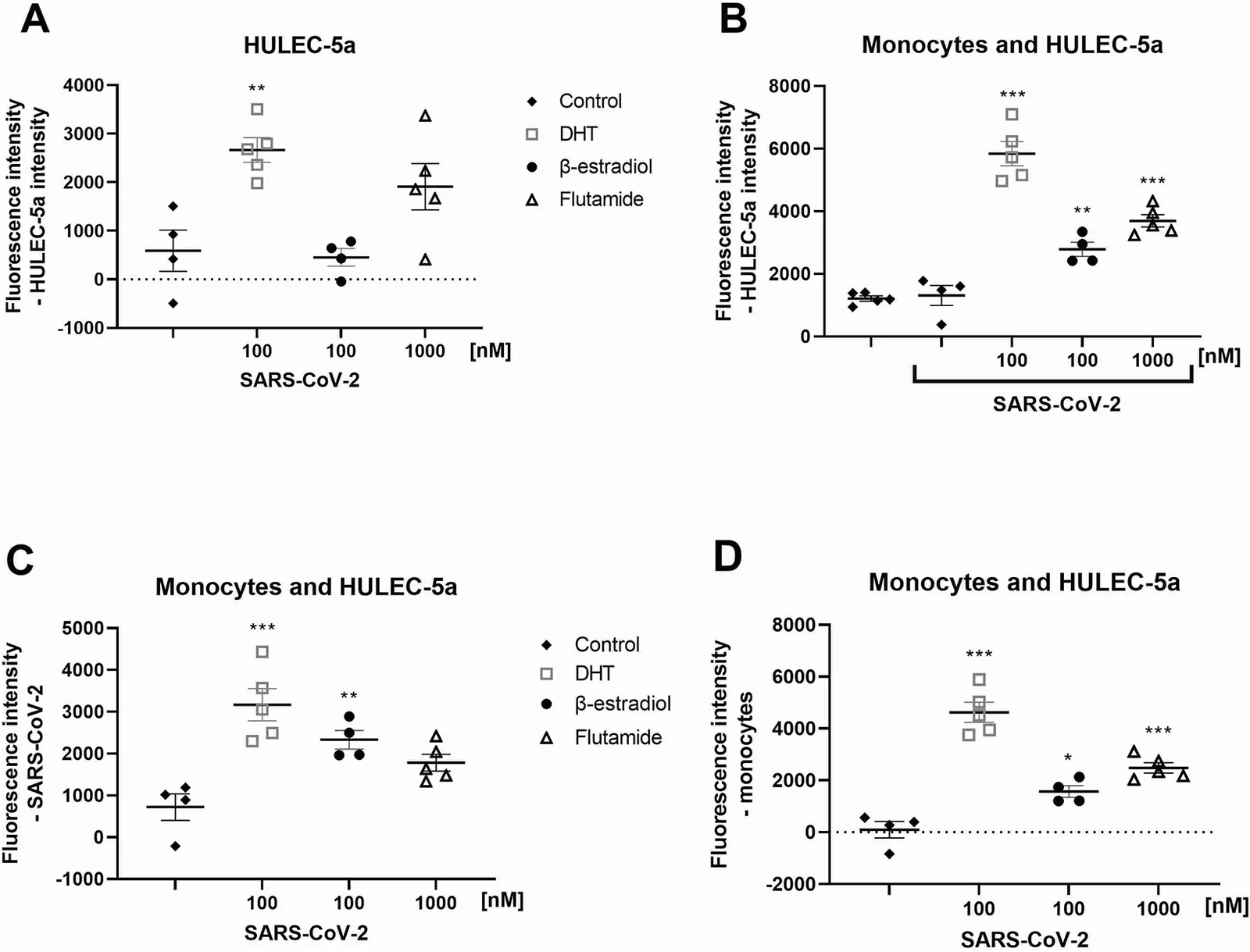
Trans-endothelial migration assay of monocytes exposed to SARS-CoV-2. Lung microvascular endothelial cells (HULEC-5a) were cultured for 24h on the transwell insert and stimulated with dihydrotestosterone (DHT, 100 nM), β-estradiol (100 nM), flutamide (1000 nM), or non-treated (Control) in combination with SARS-CoV-2 (MOI = 0.001). Monocytes from COVID-19 patients were added to the upper chamber and allowed to migrate for 24 h. Fluorescence intensity indicated **(A)** SARS-CoV-2 exposed HULEC-5a; and migration of monocytes through SARS-CoV-2 exposed **(B)** HULEC-5a; **(C)** HULEC-5a subtracted by the intensity of HULEC-5a exposed to SARS-CoV-2 [graph **(A)**]; **(D)** HULEC-5a subtracted by the intensity of only monocytes (graph **(B)**, first column). Migrated monocytes and SARS-CoV-2 exposed HULEC-5a were detached, lysed, and fluorescence intensity was measured. Values are mean ± SEM (*n* = 4-5). *p < 0.05, **p < 0.01, ***p < 0.001 vs. SARS-CoV-2 only exposed Control.

## 4 Discussion

Biological sex has been recognized as an important determinant of COVID-19 severity, yet the molecular basis underlying sex-dependent regulation of inflammatory and endothelial responses to SARS-CoV-2 remains incompletely understood. In this study, we investigated selected sex steroid receptor expression, inflammatory signaling pathways, and endothelial–immune interactions in endothelial cells and primary immune cells exposed to SARS-CoV-2. Our findings demonstrate that SARS-CoV-2 differentially modulates *ESR1* and *AR* expression in a cell-specific manner and induces distinct patterns of NF-κB, STAT3, and p38 MAPK activation. DHT stimulation enhanced inflammatory signaling in immune cells and promoted pro-inflammatory endothelial responses and transendothelial monocyte migration, whereas β-estradiol predominantly attenuated inflammatory signaling, particularly in neutrophils. Together, these findings provide mechanistic insight into the contribution of sex steroid signaling to inflammatory responses during SARS-CoV-2 infection and support further investigation of hormone-regulated pathways as potential modulators of COVID-19-associated vascular and immune dysfunction.

We observed that SARS-CoV-2 reduced the expression of *ESR1* in endothelial cells and of *AR* in neutrophils. In contrast, *ESR1* expression was increased in neutrophils and monocytes from patients with COVID-19. Previous studies have shown that sex steroids significantly influence immune responses, endothelial homeostasis, and susceptibility to severe COVID-19 (Kumar et al., 2021; Takahashi et al., 2020; Samuel et al., 2020). Estrogen signaling has generally been associated with anti-inflammatory and vasoprotective effects, including maintenance of endothelial integrity and attenuation of excessive cytokine production (Youn et al., 2021; Li et al., 2022). Therefore, suppression of *ESR1* expression in endothelial cells by SARS-CoV-2 may contribute to endothelial dysfunction, vascular inflammation, and thrombotic complications of severe COVID-19 (Solis et al., 2022; Aguilar-Pineda et al., 2021; Costa et al., 2022). In contrast, the increased *ESR1* expression observed in circulating innate immune cells from patients with COVID-19 may represent a compensatory immunoregulatory response to systemic inflammation.

Our results further demonstrated activation of NFκB, STAT3, and p38 MAPK signaling pathways in monocytic cells following SARS-CoV-2 exposure, with additional enhancement by androgens. Activation of these pathways has been widely implicated in COVID-19-associated hyperinflammation and cytokine dysregulation (Nie et al., 2023; Phuong Le et al., 2026; Faist et al., 2023). NFκB signaling is considered a central mediator of SARS-CoV-2-induced inflammatory responses and contributes to the production of IL-6, TNF-α, and other pro-inflammatory cytokines associated with cytokine storm (Patra et al., 2020; Li et al., 2021). Similarly, excessive STAT3 activation has been linked to severe COVID-19, immune dysregulation, and thromboinflammatory complications (Matsuyama et al., 2020). Activation of p38 MAPK signaling has also been associated with SARS-CoV-2 infection and may promote the release of inflammatory cytokines, endothelial activation, and tissue injury (Bouhaddou et al., 2020). Our findings suggest that androgenic signaling may amplify these inflammatory cascades in monocytes, potentially contributing to the sex-related differences in COVID-19 severity reported in clinical studies (Alwani et al., 2021; Niessen et al., 2022).

Interestingly, SARS-CoV-2 inhibited STAT3 signaling in endothelial cells, indicating that viral effects on inflammatory signaling are highly cell-specific. Although STAT3 activation is generally associated with inflammation, endothelial STAT3 may also exert protective effects by regulating vascular integrity and cellular survival (Jafarzadeh et al., 2021). Therefore, inhibition of endothelial STAT3 signaling by SARS-CoV-2 could contribute to endothelial dysfunction and increased vascular permeability observed in severe disease.

In neutrophils obtained from patients with COVID-19, SARS-CoV-2 and β-estradiol inhibited NFκB signaling, whereas SARS-CoV-2 simultaneously activated p38 MAPK signaling. These findings indicate differential regulation of inflammatory pathways within neutrophils during COVID-19. A previous study has shown that estrogen may exert context-dependent immunomodulatory effects, via estrogen receptors, by suppressing NFκB activation and inflammatory genes (Xing et al., 2012). Nevertheless, persistent p38 MAPK activation may sustain neutrophil activation despite partial NFκB inhibition, thereby contributing to oxidative stress, neutrophil extracellular trap formation, and tissue damage associated with severe COVID-19 (Cesta et al., 2023; Langereis et al., 2010). G protein-coupled estrogen receptor 1 (GPER1), a membrane-associated G protein-coupled estrogen receptor, is also expressed in human monocytes and neutrophils and can regulate neutrophil inflammatory responses through cAMP/PKA/CREB and MAPK signaling pathways (Rodenas et al., 2017). Despite negligible levels in monocytes, *ESR2* expression has been reported in endothelial cells and may contribute to estrogen-mediated vascular and inflammatory responses (Brundin et al., 2021; Frump et al., 2018). In contrast to β-estradiol, DHT promoted activation of NFκB and p38 MAPK in monocytes derived from patients with COVID-19. These findings are consistent with previous reports demonstrating that androgen signaling can enhance inflammatory responses and increase expression of SARS-CoV-2-associated host factors (Samuel et al., 2020; Zhu et al., 2022). Androgen-mediated amplification of NFκB and MAPK pathways may therefore contribute to exaggerated innate immune activation and heightened inflammatory responses in male patients with severe COVID-19.

A limitation of this study is that sex-stratified analyses were not uniformly performed across all experiments involving primary immune cells because of the limited availability of freshly isolated samples. In addition, the use of HULEC-5a cells, which were derived from a single male donor, limits the assessment of sex-dependent differences in endothelial responses and receptor expression. Future studies using quantitative and functional characterization of sex steroid receptor signaling will be important to further define the contribution of cellular sex and receptor-specific pathways to hormone-mediated responses to SARS-CoV-2.

The present findings may have broader implications for other inflammatory and vascular disorders in which sex steroids contribute to disease susceptibility and progression. Sex-dependent regulation of endothelial function, cytokine production, leukocyte activation, and thrombosis underscores the importance of sex hormones as modulators of inflammatory and vascular homeostasis (Mauvais-Jarvis et al., 2020; Klein and Flanagan, 2016). Estrogen generally preserves endothelial integrity and limits excessive inflammatory signaling (Youn et al., 2021; Li et al., 2022), whereas androgen signaling may enhance innate immune activation in specific pathological contexts (Samuel et al., 2020; Lakshmikanth et al., 2024). The differential regulation of NFκB, STAT3, and p38 MAPK pathways observed in this study therefore may reflect fundamental mechanisms of sex hormone-mediated immunoregulation that extend beyond SARS-CoV-2 infection. These findings support further research into sex steroid-dependent inflammatory signaling as a potential therapeutic target in a broader spectrum of immune-mediated and vascular diseases.

Importantly, DHT increased trans-endothelial migration of monocytes from patients with COVID-19 and promoted detachment of SARS-CoV-2 exposed endothelial cells, suggesting that androgenic signaling may facilitate vascular inflammation and leukocyte recruitment. Endothelial dysfunction and enhanced monocyte transmigration are key events in COVID-19-associated immunothrombosis and vascular injury (Bonaventura et al., 2021). Enhanced leukocyte trafficking across the endothelial barrier may promote tissue infiltration by inflammatory cells and aggravate local cytokine production. Our findings therefore support the possibility that androgen-responsive pathways contribute to inflammatory amplification, endothelial barrier disruption, and vascular complications during SARS-CoV-2 infection. However, the specific receptor mechanisms underlying these effects remain to be established, as comprehensive protein-level characterization and receptor-specific functional studies were beyond the scope of the present study.

## Data Availability

The raw data supporting the conclusions of this article will be made available by the authors, without undue reservation.
